# Beliefs and Risk Awareness on Medications Among Pregnant Women Attending the Antenatal Care Unit in Ethiopia University Hospital. Overestimating the Risks Is Another Dread

**DOI:** 10.3389/fpubh.2020.00028

**Published:** 2020-03-03

**Authors:** Yonas Getaye Tefera, Begashaw Melaku Gebresillassie, Amanual Getnet Mersha, Sewunet Admasu Belachew

**Affiliations:** ^1^Department of Clinical Pharmacy, School of Pharmacy, College of Medicine and Health Sciences, University of Gondar, Gondar, Ethiopia; ^2^Department of Obstetrics and Gynecology, School of Medicine, College of Medicine and Health Sciences, University of Gondar, Gondar, Ethiopia

**Keywords:** belief, risk awareness, medications, pregnancy, Ethiopia

## Abstract

**Background:** Most studies on drug use during pregnancy were generally focused on potential teratogenic effects. However, beliefs and risk awareness of medications can also influence medication use and fetal well-being.

**Objective:** This study aimed at assessing the risk awareness and beliefs on medication use among pregnant women attending antenatal care unit in an Ethiopian university hospital.

**Method:** A cross-sectional study was employed in pregnant women who were attending for antenatal care service at Gondar University Referral Hospital from March 15, to April 15, 2016. A pretested structured interview questionnaire adopted from the Beliefs About Medicines Questionnaire (BMQ) was used for data collection. Chi-square test and binary logistic regression were used to identify possible predictors influencing the outcome variables.

**Result:** Of the 423 women approached, 384 agreed to complete the questionnaire (90.8% response rate), and the mean age of the participants was 27.22 ± 5.5 years. More than two-thirds of the respondents had two to three (46.1%) or greater than three (25.8%) pregnancy histories. A third and nearly half (45.6%) of the respondents were on the first trimester and second trimester of their gestational age, respectively. The majority (70%) of pregnant women thought all drugs are harmful if taken during pregnancy. Only few (4.2%) of the participants did not mind taking drugs without professional advice. Most (90%) of the respondents were not willing to take drugs without professional advice. Pregnant women who came from rural areas had 25% less likelihood to self-medicate, with an adjusted odds ratio of 95% CI, 0.75 (0.37, 0.96).

**Conclusion:** In this study, overestimated and exaggerated beliefs of medication risks during pregnancy are a concern, though cautious drug use is necessary and warranted. Adequate counseling has to be provided by physicians, pharmacists, and other healthcare professionals to change pregnant women's conservative attitudes and misinformed beliefs on medication risk.

## Introduction

The safety of drug use during pregnancy has gained special attention after the thalidomide incident five decades ago. Thalidomide caused thousands of babies worldwide to be born with malformed limbs. This disastrous incident offered a tragic example of the harmful effects of drugs during pregnancy, which brought a paradigm change in pharmaceutical regulation and cautious drug use. Pregnant women should receive communication and have awareness of the benefits and potential risks of medication for an optimal therapeutic outcome (1, 2).

Various reasons made it challenging to assess the true fetal risk caused by medicine use during pregnancy. Increasing prevalence of multiple drug use, which might complicate proof of causality, and difficulties in recalling the medication used before and during the first months of pregnancy are among the mentioned barriers. Furthermore, the low incidence of major congenital malformations in the general population is estimated to be 1 ± 5%, which makes it problematic to identify fetal risk caused by medicines (3–5). Drug use throughout pregnancy brings a particular apprehension due to the probable teratogenic effects of some medications on the fetus and physiologic changes in the mother (6, 7). It is not possible to avoid drug therapy, and it may be dangerous since some women require ongoing pharmacologic treatment (7). Therefore, pharmacological therapy might be essential for both the fetus and the mother. In such a case, pregnant women should be aware of the possible risks and benefits of drug use by discussing with physicians and other healthcare providers (7).

Pregnant women's knowledge about medications has direct relationships with whether they take prescription, over-the-counter (OTC), or herbal medications during their pregnancy (8). Therefore, their medication adherence was highly influenced by their attitude and beliefs on medication use. Pregnancy is also a period where changes in pharmacokinetics become pronounced, and drugs might cross the placenta and reach the fetus. In this regard, many women do not adhere to their medications, because of the negative image embraced by society and even also by health professionals (9–12). Besides the risk caused by the teratogenic drug exposure to the fetus, there is also jeopardy associated with misinformation about the teratogenicity of drugs. This might lead to unnecessary abortions or the evasion of needed therapy, which ultimately affects the health of the mother and the fetus (9). There is abrupt discontinuation of medication use during pregnancy caused by misinformation about fetal well-being. Effective counseling and reassuring advice from counselors led many women to restart their pharmacotherapy during pregnancy, which continued throughout breastfeeding (13).

Although some pregnant women may have sufficient knowledge about the high-risk medication in pregnancy, there is a potential “general fear” toward all medications. The uncertainty in medication use during pregnancy might result in serious consequences such as termination of a wanted pregnancy, reluctance to use drugs leading to non-adherence, and preference for herbal and self-medication practices (14). On many occasions, correct information about medication risks will help to reduce unnecessary worry by the people. The most appropriate approach to address those issues is through the provision of targeted education and continuous dissemination of evidence-based information by professionals (5).

Most studies traditionally focused on the potential teratogenic effects of drugs. However, the attitude, risk awareness, and knowledge of pregnant women regarding medications may also affect medication adherence and fetal well-being. Education level influences women's perception of medication risks during pregnancy. Women with a low literacy level perceive medication use during pregnancy as more harmful (15). The Beliefs About Medicines Questionnaire (BMQ) was developed in an effort to understand people's perception of medicines. The BMQ was widely used to evaluate opinions toward medicine among people with no known disease condition or patients with specific disease conditions (16).

Reduced health-seeking behavior, late initiation of antenatal care, low level of maternal literacy, unfortunate access to health facilities, lack of comprehensive information, and poor regulation of prescription and OTC drugs were the aggravating factors of inappropriate drug use among pregnant women in developing counties (17). Drug information centers are crucial sources of medication information for pharmacists, nurses, and physicians to counsel pregnant women. Providing proper information could reduce the misinformation and unnecessary termination of pregnancies and promote rational drug therapy (18).

To the best of our knowledge and in our literature search, no study had been conducted in Ethiopia to explore the beliefs and risk awareness of pregnant women on medications that could affect the medication intake behavior during gestation. This study aimed at assessing pregnant women's risk awareness and beliefs on medications and identifying the associated factors during pregnancy in an Ethiopian university hospital.

## Methods and Materials

### Study Design and Setting

A cross-sectional study was employed in the antenatal care unit of Gondar University Referral Hospital (GURH), which is located in the northwest of Ethiopia. The source population was all pregnant women who came for antenatal care service at GURH. All pregnant women aged 18 years and above available during the data collection period between March 15, and April 15, 2016, and those willing to participate were the study participants. Pregnant women who had a disability (could not hear, could not see, and could not speak) and those with psychiatric illnesses were excluded from the study.

### Questionnaire

The questionnaire used in this study was adopted from the BMQ and the recently updated non-modified version of it (19, 20). Medication risk awareness was partly measured by using the modified BMQ and was also assessed using an approach to measuring risk awareness by developing additional questions such as mentioning the names of drugs to be avoided during pregnancy. Questionnaires to evaluate pregnant women's risk awareness and specific belief on drug use during the gestational period were partly adapted from various literature, the original BMQ, and the study by Noha et al. on pregnant women in Saudi Arabia (15), since the BMQ does not include those modified items in the context of pregnancy. We adapted to the pregnant women by evaluating the content validity and testing for sensitive questions during the pretest.

The questionnaire was pretested on 50 pregnant women prior to gross data collection, which was excluded from the final analysis. Relevant modifications were instituted, and culturally sensitive and leading questions were avoided in the questionnaire. For instance, “Did you have an abnormal child before due to medication use during pregnancy?” was removed from the medication risk awareness section based on the pretest findings. The questionnaire was prepared in English, translated to Amharic, and back-translated into English in order to ensure meaning consistency.

The reliability (psychometric property) of the tool was evaluated, and it demonstrated a Cronbach alpha value of 0.829. The content validity of the questionnaire was evaluated by a team of experts, including Obstetrics and Gynecology resident physicians, health information experts, and clinical pharmacists. The final questionnaire was categorized into two parts. Part one included sociodemographic items (age, residence, income, occupation, level of education, and obstetric history). Part two included questions evaluating the general beliefs on medications, specific beliefs on drug use during pregnancy, and risk awareness of medications used during gestation.

### Data Collection and Analysis

Data were collected by three well-trained midwives and two of the authors of the study through an interviewer-administered structured questionnaire. Data were checked for completeness and entered and analyzed by using Statistical Packages for Social Sciences (SPSS) version 20. Frequencies, percentages, and binary logistic regression were used to analyze and identify predictor variables that would alter outcome variables. *The covariates included for the adjusted odds ratio were based on the purposeful selection of variables during the crude odds ratio univariate analysis generating a p-value of*<*0.3 as cutoff point for incorporation in the multivariable adjusted odds ratio to achieve a final parsimonious model*. A *P* < 0.05 and 95% confidence interval (CI) were used as cutoff points for determining the statistical significance of associations among different variables.

### Operational Definitions

Risk awareness: The pregnant women's knowledge about medication use and assumptions of fetal risks caused by drug use during pregnancy.

Belief: Pregnant women's perception about medication intake, harms, and benefits during pregnancy.

Teratogenic: Used to describe the substances that can induce developmental, structural, or functional abnormality in the fetus.

Self-medication practice: The use of medicines without professional advice or non-prescription drug use during pregnancy.

## Results

### Sociodemographic Characteristics of the Study Participants

During the 1-month data collection period among the 423 women approached, 384 agreed to complete the questionnaire (90.8% response rate) and were included in the final analysis. The mean age of the participants was 27.22 ± 5.5 years. Half of the respondents (49.7%) were orthodox Christians, followed by Muslims (34.1%). Most (91.4%) of the participants were married, and more than half (53.4%), them were housewives. More than a third (38.3%) of the respondents were illiterate, and 2.9% of them only attended tertiary (college and university) education. More than the two-thirds of the respondents had a history of two to three pregnancies (46.1%) or greater than three pregnancies (25.8%). One-third and nearly half (45.6%) of the respondents were on the first trimester and second trimester of gestational age, respectively (Table 1).

**Table 1:** Socio-demographic characteristics of the respondents (*N* = 384) at GURH, 2016.

**Variables**	**Frequency (%)**
Religion	
Orthodox	220 (49.7)
Muslim	131 (34.1)
Protestant	31 (8.1)
Others	2 (0.5)
Ethnic group
Amhara	238 (61.9)
Tigre	139 (36.2)
Qimant	18 (4.7)
Others	7 (1.8)
Marital status	
Single	12 (3.1)
Married	351 (91.4)
Divorced	14 (3.6)
Widowed	7 (1.8)
Occupational status	
Student	19 (4.9)
Government employee	55 (14.3)
Merchant	63 (16.4)
Farmer	18 (4.7)
House wife	205 (53.4)
Self-employed	24 (6.3)
Level of education	
Illiterate	147 (38.3)
Primary school (1–8)	112 (29.2)
High school (9–12)	114 (29.7)
College and university	11 (2.9)
Monthly income in ETB	
<500	24 (6.3)
500–1,499	45 (11.7)
1,500–2,499	195 (50.8)
2,500 and Above	120 (31.3)
Residence	
Urban	279 (72.7)
Rural	105 (27.3)
The number of pregnancies	
First	108 (28.1)
2–3	177 (46.1)
More than 3	99 (25.8)
Duration of Gestation	
First	128 (33.3)
Second	175 (45.6)
Third	81 (21.1)

*ETB, Ethiopian Birr*.

### Risk Awareness About Medications

The majority (70%) of pregnant women thought all drugs are harmful if taken during pregnancy. In this regard, 346 (90%) of the respondents were not willing to take drugs without professional advice. Abnormality of the child (26%), abortion (24.8%), and death of the fetus (16.8%) were the most frequent reasons of the respondents for not taking medicines during pregnancy without professional advice. Most of the mothers (92%) did not know the medications that should be avoided during pregnancy. However, 6% of pregnant women appropriately mentioned drugs (misoprostol, warfarin, and tetracycline) avoided during pregnancy (Table 2).

**Table 2:** Risk awareness among pregnant women toward medications (*N* = 384) at GURH, 2016.

**Questions**	**Frequency (%)**
Do you think that all drugs are harmful during pregnancy?	
Yes	269 (70)
No	89 (23.2)
I don't know	26 (6.8)
Do you to take medications during pregnancy without professionals' advice?	
Yes	16 (4.2)
No	346 (90)
Not sure	22 (5.8)
Reasons of participants why they do not like to take medications during pregnancy without professionals advice (*n* = 346)	
Abortion	86 (24.8)
Abnormality to the child	90 (26)
Death to the fetus	58 (16.8)
Death to the mother	37 (10.7)
Death to both the fetus and mother	37 (10.7)
I don't know	38 (11)
What is the critical time for drug use during pregnancy?	
First trimester	94 (24.5)
Second trimester	128 (33.3)
Third trimester	162 (42.2)
Do you know the main drugs that should be avoided during pregnancy?	
No	353 (92)
Yes	31 (8)
Amoxicillin	4 (1)
Tetracycline	19 (5)
Paracetamol	4 (1)
Misoprostol	2 (0.5)
Warfarin	2 (0.5)

**Figure 1:**
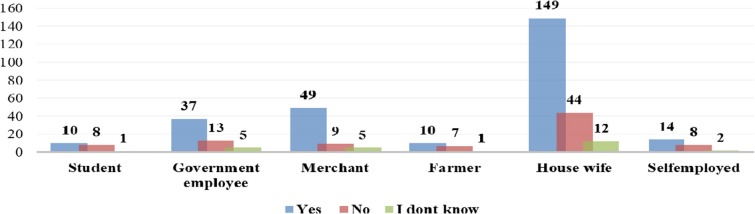
Distribution of patients' perception of drugs been harmful during pregnancy by occupation status of survey respondents at Gondar University Referral Hospital, 2016.

Occupation was the only sociodemographic variable found to be associated with the response for a question asking whether drugs are harmful during pregnancy or not. A high proportion of women who were merchants and housewives responded that drugs are harmful during pregnancy (Figure 1).

Of the pregnant women, 1.3% (five mothers) only had an abnormal child in their previous birth. Autism (1), blindness (1), hearing loss (1), and structural deformity (2) were reported, but none of the women related these incidents with drugs that they had taken during their past pregnancy.

### Sources of Information

**Figure 2:**
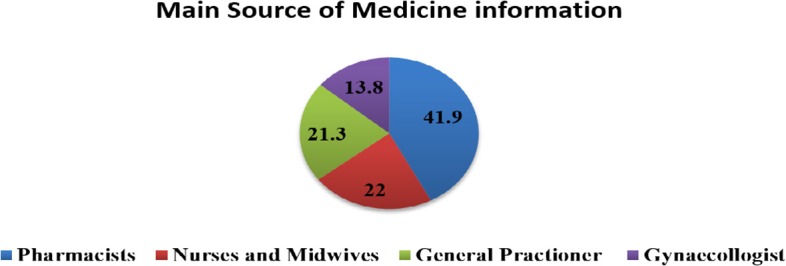
Main Source of information about dispensed medication for pregnant women at Gondar University Referral Hospital, 2016.

Only 6.8% of the pregnant women checked the leaflet of their dispensed medication. More than two-thirds (69%) of the pregnant women reported that they got adequate information from the pharmacist who dispensed the prescribed medicine. Pharmacists (41.9%), general practitioner physicians (21.3%), and nurses and midwives (22%) were the main sources of dispensed medication information for the pregnant women (Figure 2).

### Self-Medication Practice

Nearly a fifth (70 women, 18.2%) of the participants responded that they took medications without a prescription while they felt sick, but all of the medications used were anti-pain medications such as diclofenac (0.3%), paracetamol (8.6%), and ibuprofen (9.3%). Pregnant women who came from rural areas had 25% less likelihood to take medications without a prescription, with an adjusted odds ratio of 95% CI, 0.75 (0.37, 0.96) (Table 3).

**Table 3:** Odds ratio of predictive variables and non-prescription drug use (*N* = 384) at GURH, 2016.

**Variables**	**Non-prescription drug use**		**OR (95% CI)**		***P*-value**
	**Yes (%)**	**No (%)**	**COR**	**AOR**	
**Occupational status**					
Student	9 (47.4)	10 (52.6)	3.42 (0.45, 5.19)	3.17 (0.08, 8.4)	0.14
Government employed	13 (23.6)	42 (76.4)	1.17 (0.57, 2.8)	0.95 (0.03,3.07)	0.23
Merchant	8 (12.7)	55 (87.3)	0.55 (0.21, 2.87)	0.40 (0.13, 2.08)	0.27
Farmer	10 (55.5)	8 (44.5)	4.75 (0.15, 66)	3.5 (0.31, 5.54)	0.07
Housewife	25 (12.2)	180 (87.8)	0.53 (0.17, 4.23)	0.24 (0.02, 2.12)	0.13
self employed	5 (20.8)	19 (79.2)	1	1	Ref
**Education level**					
Illiterate	29 (19.7)	118 (80.3)	0.29 (0.17, 2.06)	1.236 (0.81,4.06)	0.32
Primary	22 (19.6)	90 (80.4)	0.29 (0.08, 8.647)	0.18 (0.59, 9.55)	0.403
High school	14 (12.3)	100 (87.7)	0.17 (0.11, 1.49)	0.39 (0.09, 1.89)	0.345
College and university	5 (45.5)	6 (54.5)	1	1	Ref
**Residence**					
Rural	14 (13.6)	91 (86.4)	0.61 (0.25, 0.90)^*^	0.75 (0.37, 0.96)^*^	0.04^*^
Urban	56 (20.1)	223 (79.9)	1	1	Ref

* *P < 0.05. AOR, adjusted odds ratio; CI, confidence interval; COR, crude odds ratio; OR, odds ratio*.

### Beliefs About Medications

Most (91.1%) of the pregnant women disagreed that medicines do more harm than good, while 4.5% of them agreed that medicines are poisons; 29.7% of them thought doctors place too much trust in medicines (Table 4).

**Table 4:** Pregnant women general belief about medicines (*N* = 384) at GURH, 2016.

**Statements**	**Agree *N* (%)**	**Uncertain *N* (%)**	**Disagree *N* (%)**
Doctors prescribe too many medicines	52 (13.6)	10 (2.6)	332 (83.8)
Most medicines are addictive	102 (26.5)	48 (12.5)	234 (61)
Natural remedies are safer than medicines	8 (2.1)	82 (21.3)	294 (76.6)
Medicines do more harms than good	8 (2.1)	25 (6.8)	350 (91.1)
All medicines are poisons	17 (4.5)	20 (5.2)	347 (90.3)
Doctors place too much trust on medicines	114 (29.7)	53 (13.8)	217 (56.5)
If doctors had more time with patients, he/she would prescribe more medicines	131 (34.1)	36 (9.4)	217 (56.5)

Nearly two-thirds (62.5%) of the pregnant women agreed that all medicines are harmful to the fetus; 85.7% of them disagreed that natural remedies generally should be used by pregnant women, and two-thirds of them believe natural remedies should not be used without professional advice (Table 5).

**Table 5:** Pregnant women's specific beliefs about medication use during pregnancy (*N* = 384) at GURH, 2016.

	**Statements**	**Agree *N* (%)**	**Uncertain *N* (%)**	**Disagree *N* (%)**
1	All medicines are harmful to the fetus	240 (62.5)	34 (8.9)	110 (28.6)
2	If I were not pregnant, I believe it is better for the fetus to refrain from using medicines during pregnancy	47 (12.4)	36 (9.4)	301 (78.2)
3	I have a higher threshold to uses medicines more frequently during pregnancy	75 (19.4)	42 (11.2)	267 (69.4)
4	Medicine use during pregnancy saved lives of many unborn baby	120 (31.2)	153 (50)	111 (18.8)
5	Better for the fetus if I use medicine and get well than to have untreated illness during pregnancy	269 (84.7)	32 (8.1)	83 (7.2)
6	Doctors prescribe too many medicine during pregnancy	52 (13.6)	10 (2.7)	322 (83.7)
7	Natural remedies can generally should be used by pregnant women	20 (5.2)	34 (9.1)	330 (85.7)
8	Pregnant women should preferably use natural remedies during pregnancy	64 (15.3)	40 (6)	280 (78.7)
9	Pregnant women should not use natural remedies without the health care professional's advice	250 (65)	17 (16.6)	117 (20.4)

## Discussion

The aim of this study was to assess the beliefs and risk awareness of pregnant women on medication use during pregnancy. To our knowledge, this is the first study in Ethiopian women to explore the beliefs and risk awareness on medication use during pregnancy. The perception of risk and belief on medication use will have paramount importance in the pregnant women's decision to seek healthcare services and improve medication adherence. Though knowledge and informed beliefs of pregnant women regarding the harmful effect of drugs are of great importance, incorrect or insufficient perception may lead to the unjustified termination of pregnancy (5). Nearly greater than half of the pregnant women in this study mentioned abortion and abnormality of the fetus as their reasons for not using medicines during pregnancy. The beliefs held by the pregnant women also affect medication adherence and ultimately might result in a poor treatment outcome in maternal health and subsequently affect fetus well-being (10, 12, 16).

A quarter of pregnant women thought the first trimester is the critical time for drug use during pregnancy, while nearly half of them believed the third trimester is critical. It seems women understand the drugs may have harmful effect at any time during pregnancy even though the usual belief is that teratogenicity occurs during organogenesis in the first 3 months of gestational age (21, 22).

Regarding the general belief statement of this study, most of the pregnant women disagreed that medicines do more harm than good. This is imperative for women to have a positive attitude in general toward medicines that is acceptable. Among the women who had an abnormal child, none considered medications as the cause; rather they attributed it to heredity, a curse, or something supernatural, since it is believed by the majority of African women that birth defects are attributed to divine factors (23). This is similar to a Saudi Arabia study that indicated that women believe that drugs were not the reason for congenital abnormality (15). Despite the general belief they had that medicines do more good than harm, a significant majority of women believed medications are harmful to the fetus if used during pregnancy, with regard to both the risk awareness and specific belief on medications during pregnancy. These statements highlighted that most women generally believe medicines are more useful, but they perceive medicines as harmful to the fetus in the pregnancy-specific beliefs questionnaire. But, most of the pregnant women believed that medical treatment is better for the fetus than untreated illness. This made them very cautious and not willing to take medications without professional advice. This is in agreement with the study conducted in Saudi women, which revealed that 59% of women believe medicine use is harmful to the unborn baby (15). These were more often related to women who are unsure about medication use, and they often have concerns regarding the risks of drug use during pregnancy (5, 11).

Though pharmacies are not an integral part of the routine antenatal care programs in Ethiopia, most of pregnant women prefer physicians and pharmacists as their primary source of information on the drug-related issues. Among pregnant women, 41.9% consider pharmacists as their main source of information on the dispensed medication, while 43.2% of them use physicians as their main drug information source. This finding is in agreement with a study conducted in the United Kingdom and Norway, in which physicians and pharmacies were used as the main source of drug information for pregnant women (24, 25).

Most pregnant women in this study have disagreed with the use of natural remedies during pregnancy, and this was shown by the small number of natural remedy users (5.2% only). This indicated a reluctance and conservative approach to using natural remedy alternatives, unlike women in Britain, Norway, and Italy (14, 26, 27). But it is quite similar to the Noha et al. study in Saudi Arabia, where 79% of women disagreed to natural remedy use (15). The probable reasons to refrain from using herbal medicines are side effects and controversial beliefs regarding natural remedies, as safety is not well-recognized (28). This was also supported by only 2.1% of pregnant women in the present study believing that natural remedies are safer than medicines. It is reasonably acceptable, since the safety of most of the natural remedies including herbal drugs is not well-studied, and avoidance is recommended during pregnancy (17, 29). Moreover, pregnant women might be informed and seek professional advice from their healthcare provider to make decisions on an individual basis regarding natural remedies.

Among pregnant women, 18% practice self-medication with OTC analgesic medications like paracetamol and ibuprofen when they feel sick. Self-medication with OTC drugs without professional advice is not recommended due to potential safety issues during pregnancy. This is almost similar to the study conducted by Kebede et al. at the Ethiopian capital, Addis Ababa (17). Sociodemographic factors such as education and occupation may have a significant effect on patients' risk awareness and beliefs on medications (16). In this study, rural residency was the only sociodemographic factor associated with less use of medication without a prescription. Women living in rural Ethiopia did not have easy access to medicine retail outlets (pharmacy and drug shops) in the nearby area to request OTC medications, so self-medication practice in such areas would be low.

In our study, pregnant women's general beliefs on the statements “Doctors rely too much on medicines,” “All medicines are poisons,” and “Medicines do more harm than good” are almost in agreement with the survey conducted in British women. A difference was observed in the statements “If doctors had more time with patients, they would prescribe more medicines” (34.1%), “Natural remedies are safer than medicines” (2.1%), and “Most medicines are addictive” (26.5%), while 43, 19.6, and 17.4% of British women agreed, respectively, to the aforementioned statements (24). This might be attributed to the difference in drug prescribing patterns of physicians, cultural and socioeconomic differences in safety perception of natural remedies, and medicine addiction interpretation.

Generally, with the present study, pregnant women should be cautious and make informed decisions on drug and natural remedy use during their gestational period. But, the overall belief of pregnant women should not menace most medications as detrimental to the fetus, since medication treatment outweighs most of the expected non-treatment harms and drug-related adverse events that happen to the fetus and the mother.

### Limitation of This Study

This is a hospital-based cross-sectional study conducted in a relatively small population of pregnant women coming to the hospital during the 1-month study period. We are not sure that the flow of pregnant women to the antenatal care service is similar in the other months. Besides this, the sampled women who came to the antenatal care services might be informed and knowledgeable due to the frequent contact they had with healthcare professionals, which may not represent the general population of pregnant women. There is a potential recall bias to remember and mention some of the medicines used during pregnancy from self-medication practice. As this is the first study to assess beliefs and risk awareness in Ethiopian pregnant women, we recommend multicenter and large studies to be conducted for a better understanding of the belief of women in this population.

## Conclusion

In this study, pregnant women had reservations and were reluctant to use medicines during pregnancy. Though cautious drug use is necessary and acceptable during pregnancy, they overestimated the risks associated with medication use, and their beliefs on medicines were somewhat misleading. Pregnant women had much trust in healthcare professionals for advice about medication use. While it is good to have trust in healthcare professionals' advice, it is a misinformed belief to consider all-natural remedies and alternatives as harmful during pregnancy. Healthcare professionals can influence the delivery of proper medication information, which also affects pregnant women's belief. Adequate counseling has to be provided by physicians, pharmacists, and other healthcare professionals to change the women's conservative attitudes and inflated beliefs of medication risks. Appropriate health education and promotion strategies that can address traditional and cultural beliefs will be an added advantage to promote proper drug use along with addressing the safety concerns during pregnancy. Further investigation is warranted on whether these perceptions and beliefs on medications could change either before or after pregnancy.

## Data Availability Statement

The datasets for this article are part of an ongoing study and are not publicly available in order to keep the confidentiality of the information. They are available on request to the corresponding author.

## Ethics Statement

Ethical approval was obtained from the ethical review committee of the School of Pharmacy, College of Medicine and Health Sciences, University of Gondar. Written Consent from Hospital administration was obtained; informed consent from participants was also obtained prior to conducting this study. Participants were also informed that participation was voluntary and they could withdraw from the study at any stage if they desired. Participants' information was kept confidential and no personal identifier used during data collection.

## Author's Note

The abstract of this paper was presented at the 19th ISPOR Annual European Congress in Vienna, Austria, from October 28 to November 2, 2016. The abstract was published in the poster abstracts of the conference proceedings of the *Value in Health* journal.

## Author Contributions

YT conceived the study, participated in the analysis and interpretation of the findings, and drafted the manuscript and write-up. BG and AG participated in data collection and analysis. SB supervised the study and edited the manuscript. All authors read and approved the final manuscript.

### Conflict of Interest

The authors declare that the research was conducted in the absence of any commercial or financial relationships that could be construed as a potential conflict of interest.

## Abbreviations

AOR: adjusted odds ratio
BMQ: Beliefs About Medicines Questionnaire
CI: confidence interval
COR: crude odds ratio
GURH: Gondar University Referral Hospital
OR: odds ratio
OTC: over the counter
SPSS: Statistical Packages for Social Sciences.
